# Effects of Annealing and Solution Treatments on the Microstructure and Mechanical Properties of Ti6Al4V Manufactured by Selective Laser Melting

**DOI:** 10.3390/ma15051978

**Published:** 2022-03-07

**Authors:** Hassanen Jaber, János Kónya, Klaudia Kulcsár, Tünde Kovács

**Affiliations:** 1 Doctoral School on Materials Sciences and Technologies, Óbuda University, 1034 Budapest, Hungary or hassanen.jaber@utq.edu.iq (H.J.); janos@dentarttechnik.hu (J.K.); 2 Biomedical Engineering Department, College of Engineering, University of Thi-Qar, Nasiriyah 64001, Iraq; 3 Dent-Art-Technik Kft. Magyarország, 9024 Győr, Hungary; kulcsar.klaudia@dentarttechnik.hu; 4 Donát Bánki Faculty of Mechanical and Safety Engineering, Óbuda University, 1081 Budapest, Hungary

**Keywords:** selective laser melting, Ti6Al4V alloy, additive manufacturing, annealing, solution treatment, heat treatment

## Abstract

Ti6Al4V (Ti64) alloys manufactured by selective laser melting (SLM) are well known for their susceptibility to failure at a low ductility of less than 10% due to the formation of an (α′) martensitic structure. Annealing and solution treatments as post-heat treatments of α′ are considered a good way to improve the mechanical performance of SLM-manufactured Ti64 parts. In this research, the effect of heat treatment parameters such as temperature (850 °C and 1020 °C) and cooling rate (furnace and water cooling) on the microstructure and mechanical properties of the SLM Ti64 structure was investigated. It was shown that the tensile strength/ductility of the Ti64 alloy produced by SLM was determined by the post-heat treatment. The experimental results revealed that heat treatment at 850 °C followed by furnace cooling resulted in the best possible combination of ductility (13%) and tensile strength (σ_y_ = 932, σ_u_ = 986 MPa) with a microstructure consisting mainly of 78.71% α and 21.29% β. Heat treatment at 850 °C followed by water cooling was characterized by a reduction in hardness and the formation of predominantly α plus α′′ and a small amount of β. HT850WC exhibited yield and tensile strengths of about 870 and 930 MPa, respectively, and an elongation at fracture of 10.4%. Heat treatment at 1020 °C and subsequent cooling in the furnace was characterized by the formation of an α + β lamellar microstructure. In contrast, heat treatment at 1020 °C and subsequent water cooling formed semi-equiaxial β grains of about 170 µm in diameter with longer elongated α grains and basket-weave α′. Post-treatment at 1020 °C followed by furnace cooling showed high ductility with an elongation of 14.5% but low tensile strength (σ_y_ = 748, σ_u_ = 833 MPa). In contrast, post-treatment at 1020 °C followed by water cooling showed poor ductility with elongation of 8.6% but high tensile strength (σ_y_ = 878, σu = 990 MPa). The effect of aging at 550 °C for 3 h and cooling in a furnace on the microstructure and mechanical properties of the specimens cooled with water was also studied. It was found that aging influenced the microstructure of the Ti6Al4V parts, including β, α, and α″ precipitation and fragmentation or globularization of elongated α grains. The aging process at 550 °C leads to an increase in tensile strength and a decrease in ductility.

## 1. Introduction

Titanium alloys, especially Ti6Al4V (Ti64), are among the most widely used materials in biomedical engineering thanks to their high biocompatibility, high specific strength, and excellent corrosion resistance [1]. In recent years, there has been growing interest in the application of powder bed additive manufacturing (PBFAM) technology for the production of Ti64 medical implants instead of powder metallurgical, wrought, and cast processes [2,3]. This is due to the fact that PBFAM technology has the potential to produce functionally graded materials (FGMs), lattice structures (scaffolds), and complex structures (additional degrees of freedom in design) [4,5].

PBFAM technologies such as selective laser melting (SLM) and electron beam melting (EBM) are defined by ASTM [6] as technologies for manufacturing 3D components by adding and joining metal powders layer by layer based on computer software (CAD model). In general, the SLM process is preferred for Ti64 due to its high manufacturing speed, high precision, and additional degrees of freedom in designing complex structures [7,8]. SLM involves a complex interaction between the phenomena of the process (including melting and solidification) and microstructural changes or phase transformations of the material being manufactured [9]. The heating and cooling rates of the SLM technique are much higher than those of casting and welding processes and can significantly change the microstructure of Ti64 alloys [10]. Moreover, the cooling rates in PBF processes, including SLM and EBM, are sufficiently high to always produce an (alpha’) phase in Ti6Al4V printed parts compared to other AM techniques, such as DMD and WAAM, as critically discussed by Cottam et al. [11]. The main causes for the complete transformation of the (β) phase into an (α′) martensitic structure during the SLM process are the complete melting mechanism and the very high cooling rate [3]. Martensitic (α′) is a supersaturated substitutional solid solution of elements (vanadium) in a hexagonal crystal system of the (α) phase [12].

One of the main issues in the literature about SLM of Ti64 for biomedical implants is the formation of an α′ martensitic microstructure [3,13]. It has been experimentally demonstrated that SLM parts that are fully α′ martensitic have a low ductility of less than 10% [7,8,14,15,16,17]. Moreover, residual stresses are associated with the formation of an (α′) martensitic structure, resulting in lower mechanical performance [18]. Due to the highly localized heat input, short interaction times, rapid solidification, and large thermal gradients during the SLM process, thermal stresses are formed. It is well known that the fatigue performance of SLM parts can be affected by residual stresses generated by the high cooling rates and thermal gradient of the SLM process [19]. In addition, according to ASTM F13-12a [20] and ASTM F2924-14 [21], the microstructure of an implant or SLM part requires a minimum strain of 10% and an alpha (α)-beta (β) dual phase.

In [22,23,24,25,26], the authors changed the microstructure of the SLM part from (α′) to (α + β) to improve ductility and reduce internal residual stresses. The work by Xu et al. [22] revealed that the microstructure of the 90 μm thick layer is an (α+ β) structure, whereas the microstructure of the 30 μm thick layer is an (α′) martensitic structure. Ali et al. [23] presented a new principle of (α′) martensite decomposition, in which increasing the bed temperature (preheating) to 570 °C enables the decomposition of (α′) into an (α + β) structure. As indicated by Qiu et al. [24], (α′) transforms into (α) plus (β) during heat treatment by hot isostatic pressing (HIP). Vrancken et al. [25], in their work on the addition of 10 wt.% Mo to Ti64 powder during the SLM process, found that the conversion of (β) to (α′) was completely suppressed due to the reduction in the β-transus temperature from 995 °C to 900 °C. Muhammad et al. [26] investigated heat treatments of Ti64 parts produced by SLM at 950 °C, near the β-transus temperature, for 1 h, followed by furnace and air cooling. They found that the Ti64 microstructure had almost the same (α) plus (β) microstructure due to the converging cooling rate.

Consequently, there is clearly a practical need to study the microstructure evolution and mechanical performance of SLM parts after heat treatment. Heat treatments of Ti64 parts produced by SLM have not been studied in depth. Therefore, the purpose of this research was to analyze the effects of annealing and solution treatments at 850 and 1020 °C, below and above the β-transus temperature (β_tr_), on the microstructure and mechanical properties of the Ti64 microstructure manufactured by SLM. After the solution treatment, the specimens were aged (reheated) to 550 °C for 3 h. The microstructural evolution was studied by X-ray diffraction (XRD), optical and electron microscopy techniques assisted by energy-dispersive spectroscopy, and microhardness analysis. The mechanical properties were studied by tensile test.

**Figure 1:**
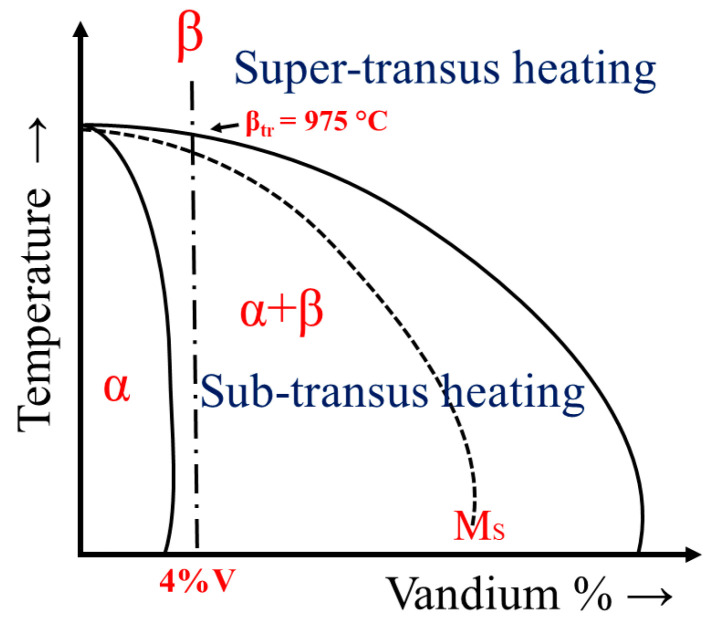
Typical equilibrium phase diagram for Ti64 alloys.

Phase transformations in Ti64 alloy can be divided into two types depending on the cooling rate: diffusive processes (nucleation and growth) and martensitic transformation (shear mechanism). Due to the fast-cooling rates of more than 410 °C s^−1^, the β phase transforms into the (hcp) crystal structure called alpha prime (α′) martensite β → α′ or into the orthorhombic structure called alpha double prime (α′′) martensite β → α′′. During the diffusive transformation, the Ti64 alloy undergoes a phase transformation in the solid state. The BCC-β phase transforms into HCP-α and BCC-β phases at slow cooling rates of less than 20 °C s^−1^ through the β-transus temperature (β_tr_), as shown in Figure 1. For this study, the β_tr_ temperature was calculated to be 975 °C according to Equation (1) [27].

β_tr_ = 882 + 21.1 [Al] + 4.2 [Sn] + 123 [O] + 23.3 [Si] − 9.5 [Mo] − 6.9 [Zr] − 11.8 [V] − 12.1 [Cr] − 15.4 [Fe] (1)

Depending on the β_tr_ temperature, the heat treatment of Ti64 can be divided into the following zones: subtransus heating (in the α + β zone) and supertransus heating (in the β zone), as shown in Figure 1. It is well known that the microstructure of Ti64 alloys can be affected by heat treatment parameters, especially by the cooling rate, time, and temperature. Therefore, based on the cooling rate, the morphologies of the α phase upon the decomposition of the β phase during cooling to below the β_tr_ temperature of Ti64 alloy can be typically expressed as primary α (α_P_), secondary α (α_S_), grain boundary α (α_GB_), alpha prime (α′) martensite, alpha double-prime (α′′) martensite, lamellar (colony), equiaxed, basket-weave (Widmanstätten), and bimodal [28,29]. The bimodal structure is a combination of lamellar and equiaxed morphologies.

## 2. Materials and Methods

### 2.1. Materials Preparation

**Figure 2:**
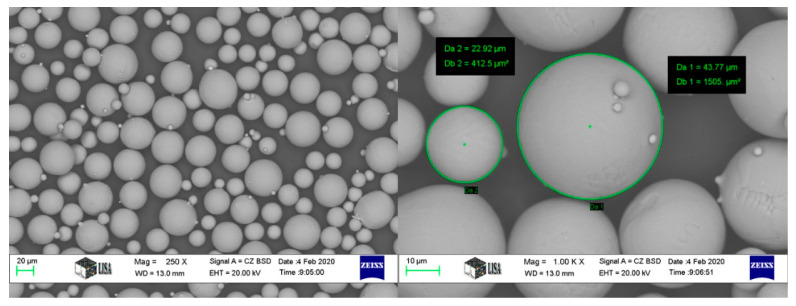
SEM micrographs show morphology of Ti64 powder at different magnifications.

**Figure 3:**
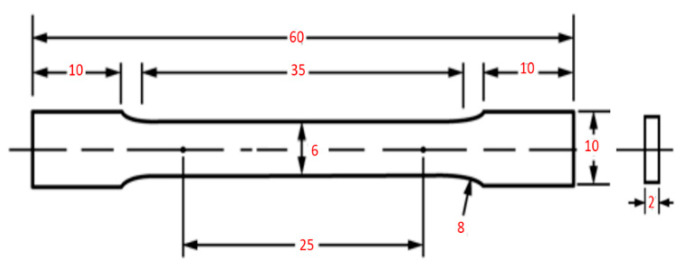
The shape and size of the tensile sample (mm).

The SLM Ti64 components were manufactured at Dent-Art-Technik Kft (Győr, Hungary) using a commercial SLM machine (Sisma mysint 100/Italy) equipped with a 200 W fiber laser and a 55 μm laser spot. In this study, Ti64 plasma-atomized spherical powder (Gr.23) supplied by (LPW Technology/UK), as shown in Figure 2, was used as the starting material. Table 1 reports the chemical composition of Ti64 powder. The size distribution is in the range of 15–45 µm (D10: 14.28 µm; D50: 25.06 µm; D90: 42.15 µm). SLM parameters were selected based on the guidelines available for the SLM machine, resulting in optimal build conditions. Throughout the process, the layer thickness, scan speed, and laser power were kept constant at 20 µm, 1000 mm/s, and 125 W, respectively. For shielding, pure argon gas was used with a flow rate of 35 L/min. The dimensions of the specimens and the configuration of the tensile test performed during the investigation are shown in Figure 3.

### 2.2. Heat Treatments 

**Figure 4:**
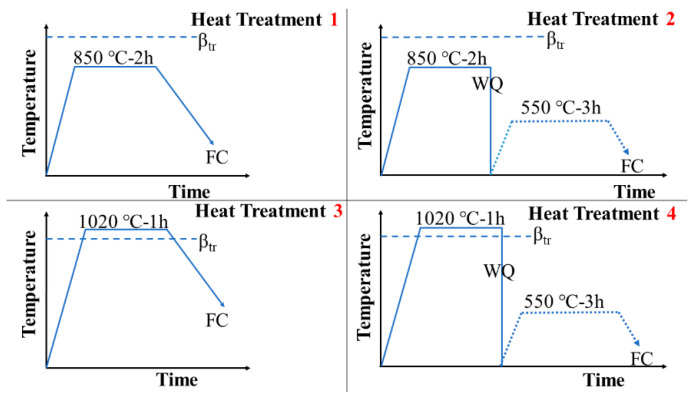
Schematic representation of heat treatment cycle used in this work.

After fabrication, four different heat treatments were performed, as shown schematically in Figure 4. In heat treatments 1 and 2, the SLM Ti64 specimens were heated to 850 ℃ (α 73% + β27%) for 2 h, and then some specimens were cooled in the furnace (FC) and others quenched with water (WC) to room temperature. In heat treatments 3 and 4, the SLM Ti64 specimens were heated at 1020 °C (β100%) for 1 h, followed by FC for some specimens and followed by WQ for others. After WQ, some of the specimens were aged at 550 ℃ for 3 h and then cooled in the furnace. HT850FC, HT850WC, HT850WC+ AG, HT1020FC, HT1020WC, and HT1020WC + AG were the designations for the corresponding heat-treated specimens at different temperatures.

### 2.3. Materials Characterization

To understand how the microstructures affect the mechanical properties, three tensile test specimens were performed at room temperature for each treatment. The tensile test was performed using a non-computerized testing machine (FORM + TEST, Model: TTM 100, Zwiefalter Straße, Germany) at a crosshead speed of 1 mm/min. All metallographic examinations of the specimens were prepared according to the standard procedure for titanium alloy metallography. Optical microscopy (OM, Neophot 2, Jena, Germany) and FEI Quanta 3D scanning electron microscope (SEM, Hitachi, Tokyo, Japan) equipped with an energy-dispersive X-ray spectrometer (EDS) were used to study the microstructure of the specimens. An EDS was used to evaluate the chemical compositions of the phases. The size of the focused electron beam is ~1 nm in the case of secondary electrons and ~2–4 nm in the case of backscattered electrons. Keller’s etchant No. 193 [30] was used to visualize the microstructure of the specimens. X-ray diffraction analysis (XRD, PhilipsX’-PertPro, Malvern, UK) with CuKα radiation was performed to analyze the phases composing all of the specimens. The tube current and tube voltage of the XRD are 20 mA and 40 kV, respectively. The Leitz-Wetzlar (Wetzlar, Germany) microhardness tester was used to determine the hardness values of the specimens. A load of 0.5 kg f and a time of 10 s were used.

## 3. Results and Discussion

### 3.1. Microstructure Investigation

The final microstructure, which determines the mechanical properties of the as-manufactured Ti64 alloy, is determined by the heat treatment parameters, mainly the cooling rate, time, and temperature. The research objectives in this study focused on heating the specimens to 850 °C or 1020 °C followed by furnace cooling (FC) or water quenching (WQ) to understand the effects of different heat treatment temperatures and cooling rates on the resulting microstructure and mechanical properties.

#### 3.1.1. Microstructure of As-Manufactured Ti6Al4V 

**Figure 5:**
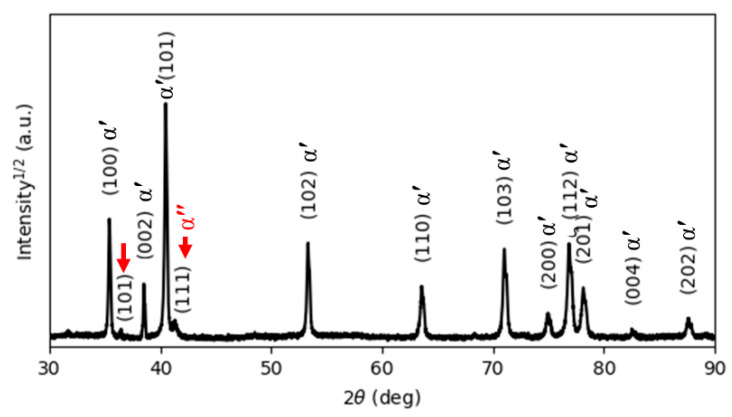
The XRD pattern of the as-printed Ti64 indexing α′ and α″ phases.

Figure 5 reports the XRD pattern of the as-manufactured specimens, in which hexagonal close-packed reflections related to the α′ martensite or α phase and a weak orthorhombic reflection related to the α″ martensite of titanium are observed. The α″ structure is indicated at the reflection plane of (111) according to Reference [31]. The presence of α″ has been found in SLM-prepared Ti64 [16,32]. Moreover, a new reflection plane (101) is seen at 2θ = 36.53°. Unfortunately, there is no clear explanation for this result. It would seem that this new reflection is related to the α″ structure.

It is well known that both α and α′ have a hexagonal structure. Therefore, it is very difficult for XRD analysis to distinguish them from each other. However, there is an important metallurgical difference between α′ and α phases, which is correlated to the content of V in the atomic structure. Due to the fast cooling rates, vanadium diffusion is inhibited; therefore, the vanadium content in the α′ phase is higher than in the α phase, leading to the development of significant deformations of the crystal structure of the α′ phase, which causes broadening of the XRD peaks. Full width at half maximum (FWHM) values were measured for the peaks around 2θ = 40.56° and 39.80°, which correspond to the diffraction of the (101) and (110) peaks of the α and β phases, respectively. Table 2 compares and summarizes the changes in the FWHM and lattice parameters of the phases in the as-manufactured state and after different heat treatments. It can be seen from Table 2 that the FWHM value of the as-manufactured specimens (0.1466) is significantly higher than that of the other heat-treated specimens because the as-manufactured specimen is mainly composed of α′ martensite. Moreover, the lattice parameters a and c of the as-manufactured specimens are 2.9324 Å and 4.6716 Å, respectively, which is in line with the lattice parameters for the α′ martensite in Reference [32].

**Figure 6:**
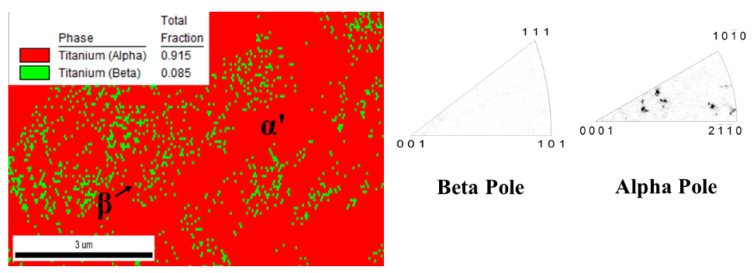
EBSD phase maps of as-printed Ti64 sample indicating the volume fractions of α′ and β phases.

No amounts of β phase were revealed by XRD analysis. In contrast, EBSD phase maps (Figure 6) of the cross-section indicate the formation of a body-centered β phase. The EBSD phase maps highlight that the volume fraction of β is about 8.5% of the Ti64 structure formed by SLM. The α′ phase and β phase are colored red and green, respectively, in the EBSD maps. It is shown that the prior β is formed by epitaxial growth during successive layer depositions [3].

**Figure 7:**
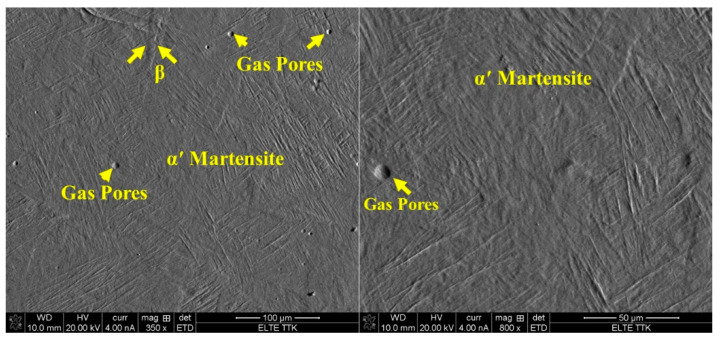
SEM images of Ti64 produced by SLM showing α′ martensite microstructure and the formation of gas pores.

**Figure 8:**
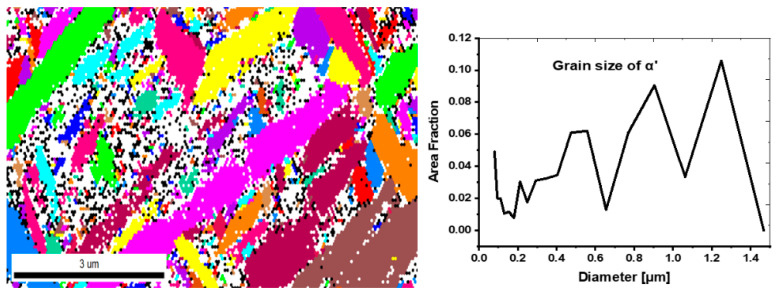
EBSD phase maps and grain size of the α′ martensite microstructure.

**Figure 9:**
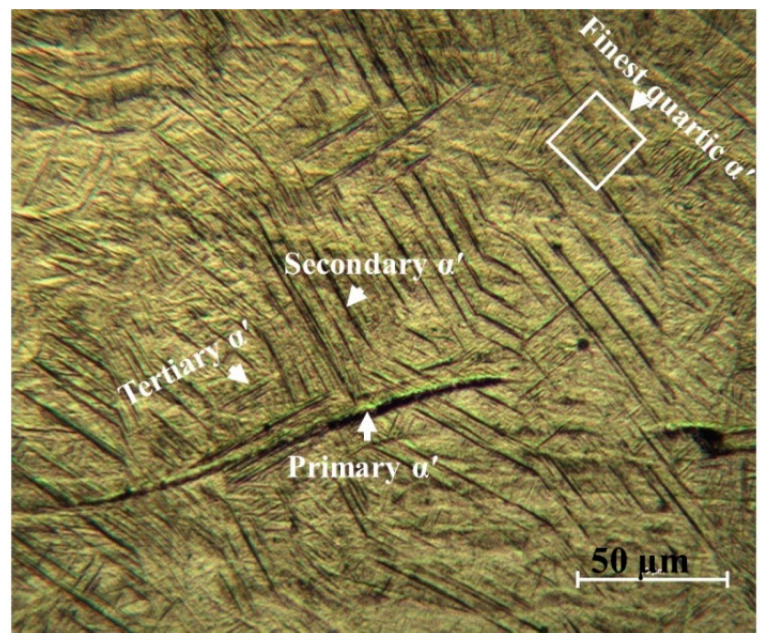
An optical micrograph showing hierarchical structure of α′ martensitic microstructure.

**Figure 10:**
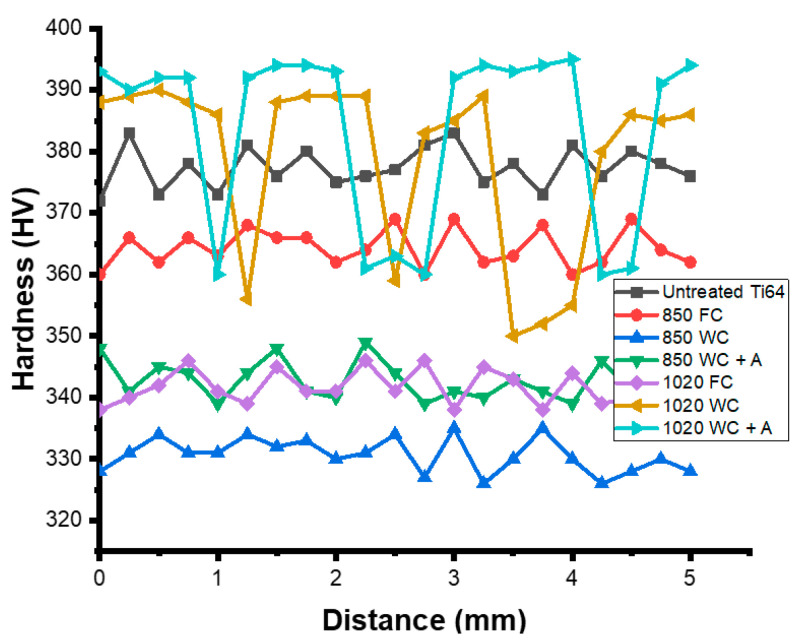
Typical hardness profile of as-printed sample and samples subjected to different heat treatments.

As was hypothesized, the SEM experiments (Figure 7) prove that the microstructure of the as-fabricated Ti64 specimens is fully α′ martensitic, with a lath morphology and a small amount of β phase. The average value of α′ lath thickness is 0.420 µm. Figure 8 shows the EBSD orientation maps and grain size of the α′ martensite microstructure in detail. Moreover, this martensitic microstructure is defined as a hierarchical structure (Figure 9), which is composed of four different types of α′ on the basis of the dimensions: primary (L = 125 µm), secondary (64 µm), tertiary (32 µm), and quartic (8 µm). These results are consistent with the findings of Yang et al. [33]. The hierarchical structure of the microstructure is recognized as a crucial feature for microstructural morphology transformation during heat treatment of as-manufactured Ti64 [34]. The formation of α′ martensite depends on the cooling rate. According to Reference [29], the critical cooling rate of Ti64 for the formation of α′ martensite in the microstructure is 410 °C s^−1^. The cooling rate during SLM of Ti64 is 10^4^ K s^−1^ [15], which is significantly higher than 410 °C s^−1^. Therefore, the formation of α′ martensite during SLM of Ti64 is not surprising. The average hardness of α′ is 377 HV (Figure 10). It is important to note that the hardness of α′ martensite is the highest among the microstructures of the other specimens, except for HT1020WC and HT1020WC + AG. 

**Figure 11:**
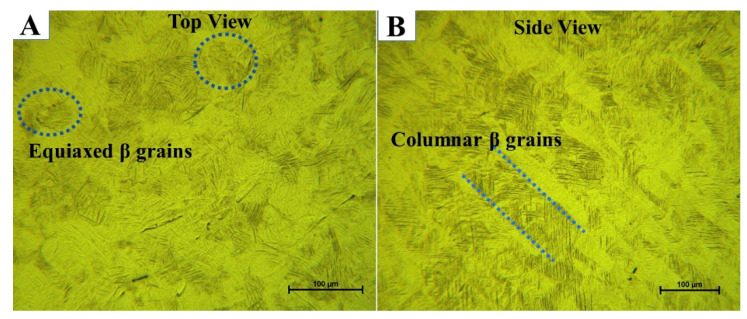
(**A**) An optical micrograph of the top view of as-printed Ti64 indicating equiaxed β grain morphologies that are fully α’ martensite. (**B**) An optical micrograph of the side view of as-printed Ti64 indicating β columnar grains.

Figure 11A,B show optical microscope images of the microstructure of as-manufactured Ti64 at low magnification of the top and side views, respectively. The microstructures do not exhibit homogeneous morphologies. In the top view, equiaxed β grains with an average diameter of 72 µm are observed to be completely interspersed with α’ martensite (Figure 11A), which can be attributed to recrystallization during the SLM process. This is completely different from what is identified in the side view (Figure 11B). The morphology in the side view consists of β columnar grains that grow epitaxially due to the re-melting and re-solidification of the material during successive layer depositions. The width of the β columnar grains is around 78 μm, which corresponds to the hatch spacing chosen for the fabrication of the specimens. Figure 7 shows a typical SEM micrograph of Ti64 produced by SLM, indicating the existence of spherical gas pores (7.5 µm) in the microstructure. Gas pores are formed by the formation of a void in the solidified pool due to trapped gas, which is not insoluble in liquid metals, resulting in a spherical void shape [10]. There are two sources of trapped gas: gas from the powder manufacturing process and inert shielding gas used with SLM.

#### 3.1.2. Microstructure of Subtransus Heat Treatments

**Figure 12:**
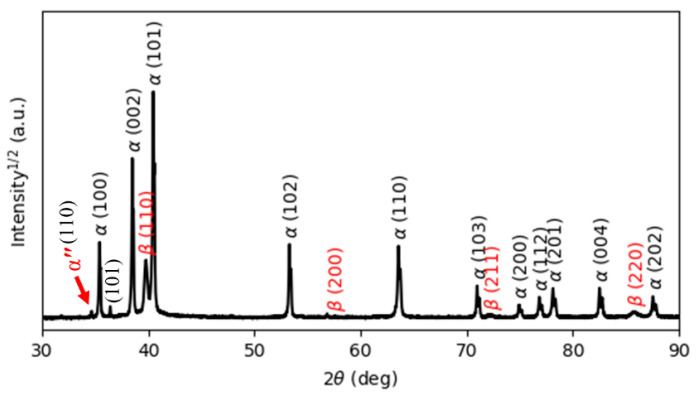
The XRD pattern of the HT850FC sample indexing α, β, and α″ phases.

**Figure 13:**
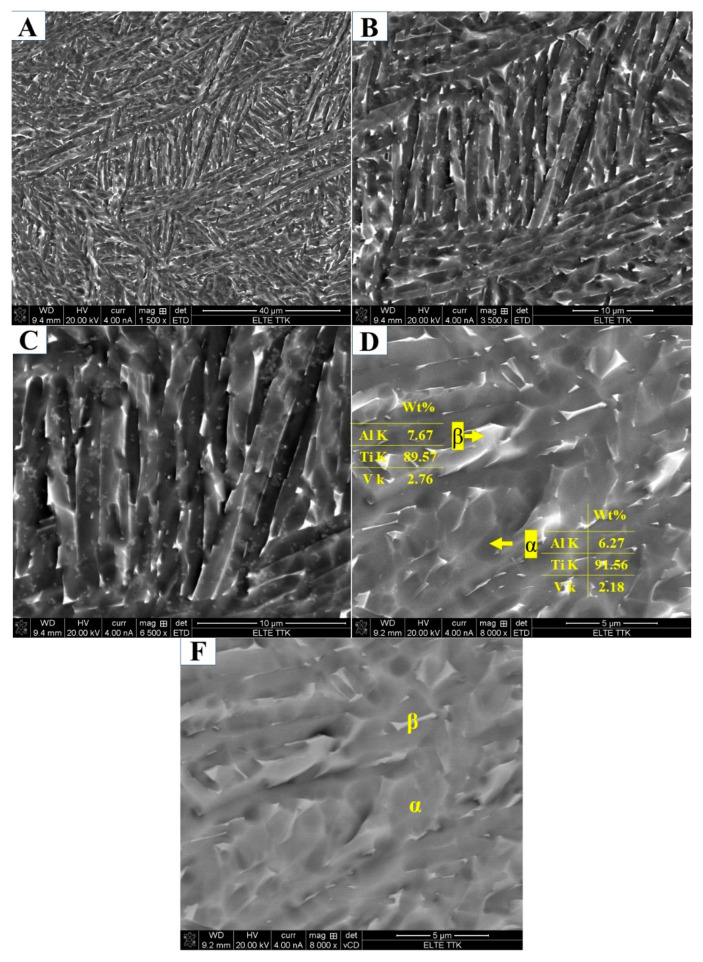
(**A**–**D**) SEM micrographs showing microstructure of the HT850FC sample at different magnifications indicating the α phase coupled with the β phase. (**F**) SEM (BSE) micrograph showing microstructure of the HT850FC sample indicating the slight chemical contrast between the α (dark phase) and β phases (lighter phase).

Figure 12 reports the X-ray diffraction pattern of the HT850FC specimen, in which reflections of the α, β, and α″ phases are observed. The β phase can be seen in the four reflection planes (110), (200), (211), and (220). It is interesting to note that the α″ structure is displayed at a new weak reflection plane of (110) (2θ = 34.73°) according to References [32,35]. This would appear to indicate that α″ is a new phase precipitated from the α′ phase during FC cooling. The reflection plane (101) at 2θ = 36.53° remains visible in the XRD pattern of the specimen after HT850FC. Figure 13 presents the microstructure of the HT850FC specimen at various magnifications, showing the α phase (dark phase) associated with the β phase (lighter phase), which EDS analysis confirms is rich in the V element (Figure 13D). The V content of the β phase (2.76 wt.%) is slightly higher than that of the α phase (2.18 wt.%). This β phase, which is poor in vanadium, is called a metastable βr phase. Backscattered electron mode (BSE, Figure 13F) confirms the slight chemical contrast between the α and β phases. It is interesting to note that the Al content in the βr phase (7.67 wt.%) is higher than that in the α phase (6.27 wt.%). The hardness values of HT850 FC averaged at 364 HV, which is lower than the hardness of the as-manufactured specimen (377 HV). This is due to the decomposition of α′ martensite into α, β, and α″ phases.

It has been shown that the α phase begins to transform into the β phase when heated to a temperature above 705 ℃ [36]. The formation of the β phase at elevated temperature can be attributed to the expulsion of vanadium atoms from the α′ phase, leading to the nucleation of the α phase along the α′ boundaries and the precipitation of the β phase in the grain boundaries of the α phase [13]. As highlighted in Table 2, the angle of the XRD peak (101) in the heat-treated specimens is shifted to lower diffraction angles compared to the as-manufactured specimen, suggesting that there is significant diffusion of vanadium in the Ti structure. The loss of interstitial or substitutional atoms (V or Al) in the hcp lattice of Ti leads to a slight increase in the c/a ratio of the α phase. All heat-treated specimens exhibit an increase in the c/a ratio of the XRD peak (101) compared to the as-manufactured specimen. From these results, it can be inferred that more vanadium was dissolved in the β phase during the heat treatments.

**Figure 14:**
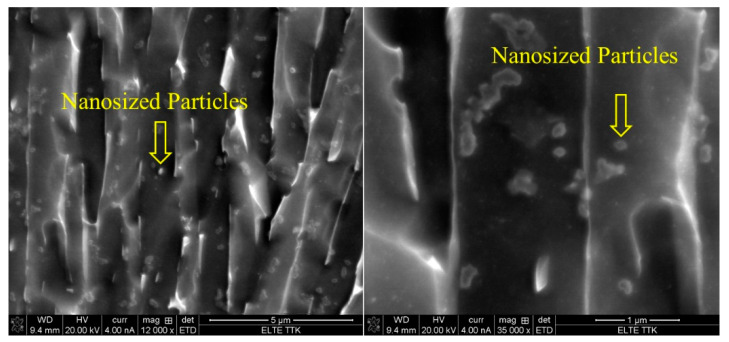
SEM micrographs at higher magnifications showing microstructure of the HT850FC sample indicating the formation of β nanosized particles dispersed on the α phase.

In this region, at temperatures of 850 ℃, below β_tr_, the α′ microstructure transforms to about 73% α and 27% β upon heating [37]. Previously, it was observed that the fine quartic α′ grains were converted to β grains, while the primary, secondary, and ternary grains of α′ were converted to α, with noticeable grain growth [34]. Cooling to room temperature reveals a mixed lamellar structure of α plus β, in which the α phase appears as fine needles. Figure 14 shows the microstructure of the HT850FC specimen at a higher magnification. It can be seen that some nanoscale particles are dispersed on the α phase. It was found that these nanoscale particles are the β phase [26].

**Figure 15:**
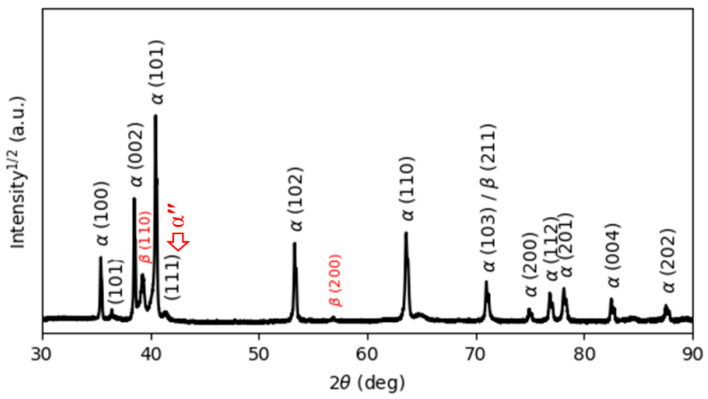
The XRD pattern of the HT850WC sample indexing α, β, and α″ phases.

**Figure 16:**
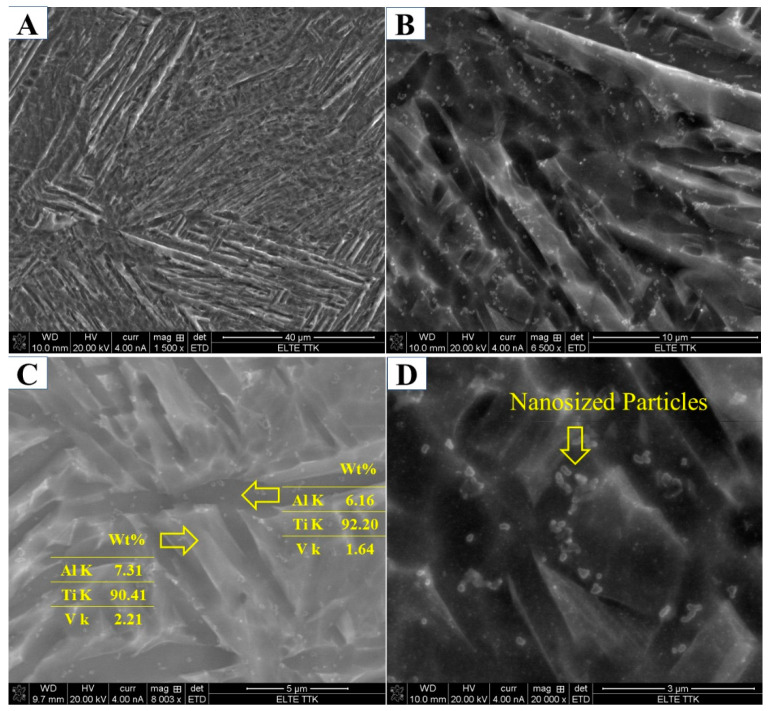
(**A**–**D**) SEM micrographs showing microstructure of the HT850WC sample at different magnifications indicating the formation of a dual-phase microstructure.

**Figure 17:**
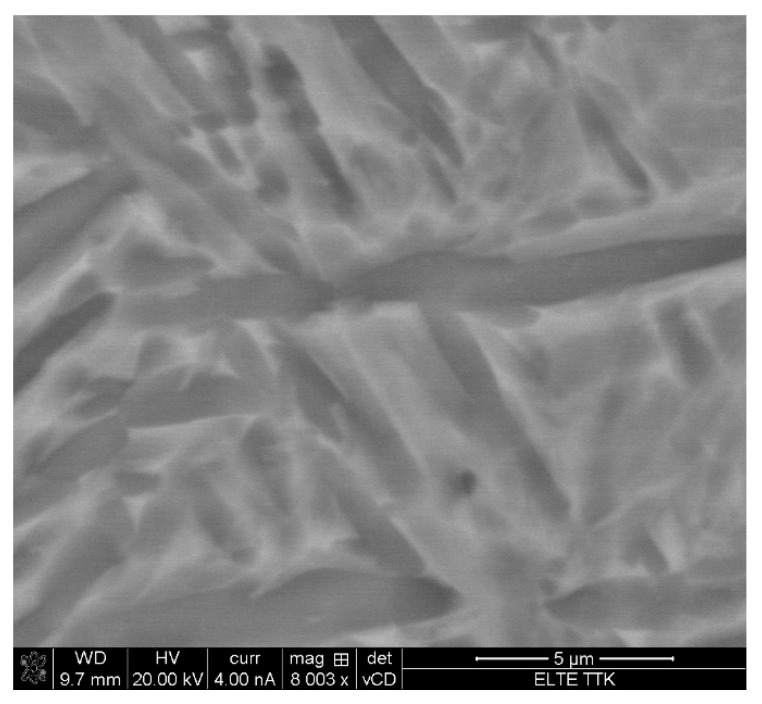
SEM (BSE) micrograph showing microstructure of the HT850WC sample indicating the considerable chemical contrast between two phases.

**Figure 18:**
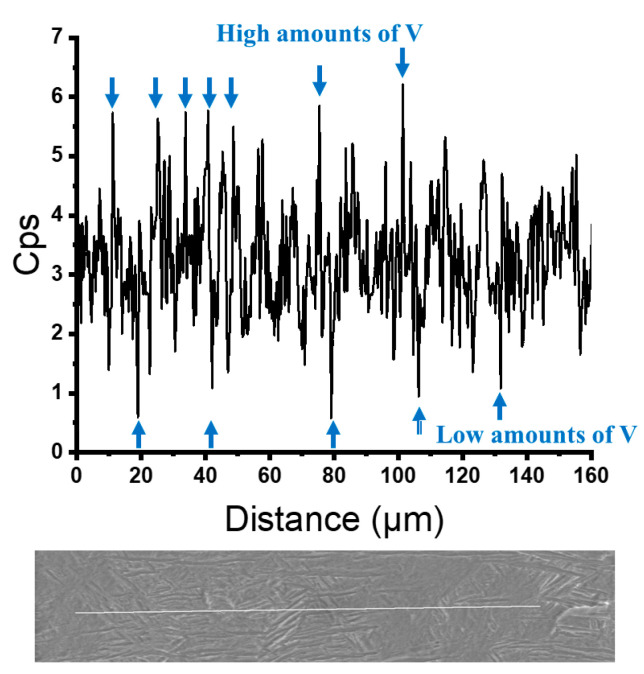
The line scan EDS results of HT850WC showing some areas are rich in V and some are poor in V.

The XRD analysis of the HT850WC specimen is illustrated in Figure 15. It can be identified that the HT850WC specimen has strong reflection peaks of the α phase. There are two reflection peaks of the β phase at (110) and (200). In addition, one reflection peak (111) of the α″ phase is identified. The reflection plane (101) at 2θ = 36.53° remains visible in the XRD pattern of the specimen after HT850WC. The full width at half maximum (FWHM) of the HT850WC specimen (Table 2) confirms that this specimen has narrower α reflections (0.094) compared to HT1020FC (0.1008), and it is broader than that of the HT850FC specimen (0.0876). These results show that HT850WC produces an orthorhombic α″ instead of α′. Figure 16A–D present the SEM micrographs of the microstructure of the HT850WC specimen at various magnifications. The EDS spot analysis of these formed phases is presented in Figure 16C. The average V content of the β and α phases was found to be 2.28 and 1.64 wt.%, respectively, which is confirmed by the backscattered electron (BSE) mode as a significant chemical contrast (Figure 17). As can be seen, this β phase is not very rich in V, indicating the formation of the metastable βr phase. In fact, the very high cooling rate in water, which prevents the diffusion of vanadium that has not reached the equilibrium state, can explain the formation of α″ martensite. It has been shown that the formation of α″ martensite depends on the V content [15]. Figure 18 is the EDS line scan across the microstructure of the HT850WC specimen. From the analysis of the composition profile, it can be deduced that some areas are rich in V and others are poor in V. The presence of high and low amounts of V can explain the formation of α″ and βr phases. The decrease in microhardness in the microstructure of the HT850WC specimens from 377 to 331 HV, as shown in Figure 10, indicates that α″ formed during water quenching. Consequently, the microstructure of the HT850WC specimen is a complex mixture of α, α″, β, and β_r_. Figure 16D shows the microstructure of the HT850WC specimen at high magnification, indicating that some nanoscale particles are dispersed on the α phase, which were confirmed to be β phase by Reference [26].

**Figure 19:**
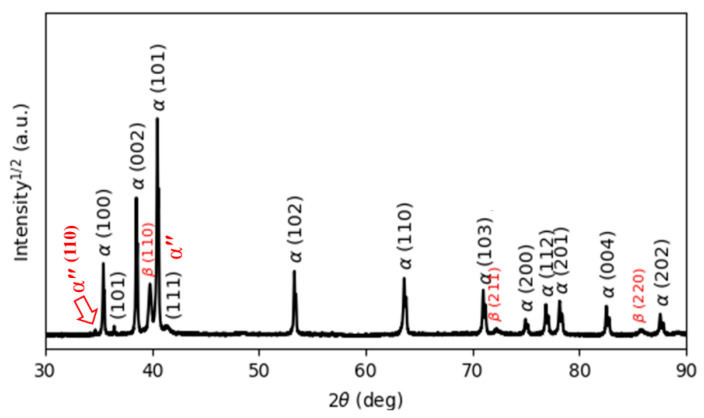
The XRD pattern of the HT850WC + AG sample indexing α, β, and α″ phases.

**Figure 20:**
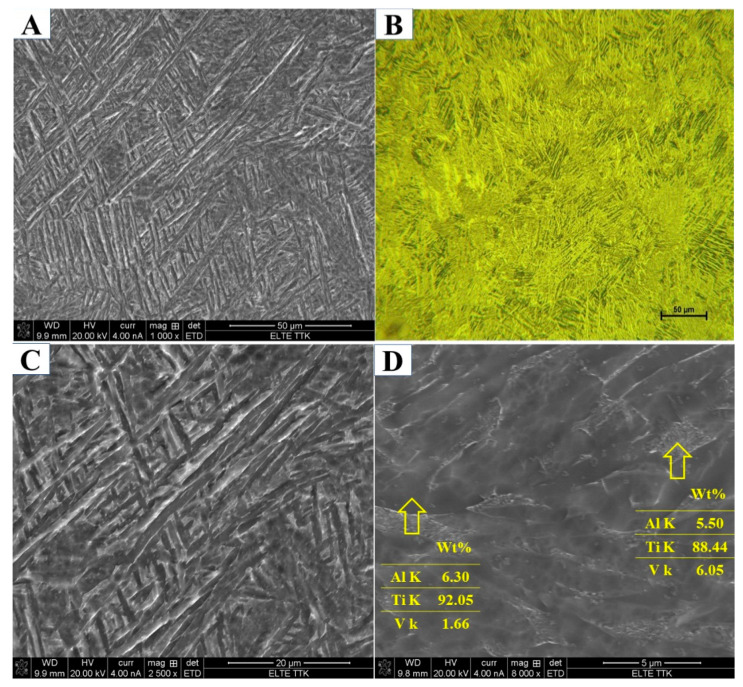
(**A**–**D**) SEM and optical micrographs showing microstructure of the HT850WC + AG sample at various magnifications.

Aging the HT850WC specimen at 550 °C for 3 h resulted in insignificant changes in the diffraction pattern (Figure 19). Three reflection peaks (110), (211), and (220) belonging to the β phase formed instead of two in comparison to the HT850WC specimen. In addition, a new weak reflection peak (110) belonging to the α″ phase is observed at 2θ = 34.73°. This indicates that new phases (β and α″) are precipitated during aging. On the other hand, the microhardness measurements of the HT850WC + AG specimen (Figure 10) indicate an increase in hardness compared to the HT850WC specimen (343 HV2 versus 330 HV2). By combining the XRD result with the microhardness result, it can be deduced that α″ partially decomposed and transformed into the α, β, and α″ phases. Figure 20A–D presents SEM and optical micrographs showing the microstructure of the HT850WC + AG specimen at various magnifications. The lattice parameters (a) of the β phase in the HT850WC specimen (a = 3.2419 Å) are higher than those in HT850WC + AG specimens (a = 3.2001 Å), as shown in Table 2. The reason for this result is the difference in chemical composition. The EDS analysis of the β phase in the HT850WC specimen (7.31 wt.% Al, 90.41 wt.% Ti, and 2.21 wt.% V) (Figure 16C) confirms that this phase has a different composition from that of the HT850WC + AG specimen (5.50 wt.% Al, 88.44 wt.% Ti, and 6.05 wt.% V) (Figure 20D). The concentration of V in the β phase of the HT850WC + AG specimen is higher than that of HT850WC. The atomic radii of V (0.132 nm) are smaller than those of Al (0.143 nm) and Ti (0.147 nm) [34]. As a result of the enrichment of the phase with vanadium, the lattice parameter of (a) decreases. As shown in Table 2, decreasing the lattice parameter of (a) causes the phase angle to move to a higher angular location, from 2θ = 39.25 to 39.80° for the (110) peak.

After aging the HT850 WC specimen at 550 °C, the average lath thickness of the α phase was increased from 1.024 to 1.342 μm, and the average volume fraction of the α phase was increased from 68.42 to 74.39%. According to the microstructure of the HT850WC + AG specimen at high magnification in Figure 20D, this considerable grain growth is due to the transformation of nanoscale particles dispersed on the α phase in the microstructure of HT850WC (Figure 16C) into the α phase. The EDS in Figure 20D seems to show that for the HT850WC + AG-treated specimens, the β phase poor in vanadium renews itself during aging by increasing the concentration of vanadium from 2.28 wt.% for the HT850WC specimen to 6.05 wt.% for the HT850WC + AG specimen due to the diffusion of vanadium from decomposed nanoscale particles into the β phase.

**Figure 21:**
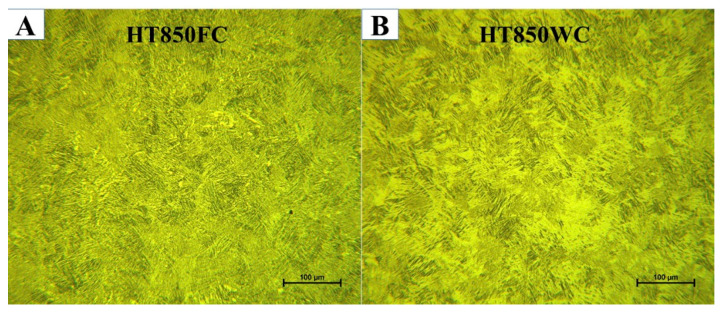
Comparison of the similarities in structure morphology between sample (**A**) HT850FC and sample (**B**) HT880WC.

Subtransus heat treatment at 850 °C for 2 h, followed by furnace cooling or water quenching, highlights a different microstructure evolution, as detailed in Figure 21. A comparison of the two figures reveals that there are significant differences in the thickness of the α phase. The average lath thickness of the α phase is 1.403 µm after furnace cooling and 1.024 µm after water quenching. Nevertheless, the lath thickness of the α phase was higher after furnace cooling than after water quenching, which can be attributed to the low cooling rates during furnace cooling, allowing the grains to grow. Therefore, it can be deduced that the cooling rate is an important factor when cooling from 850 °C, below the β_tr_. It is worth mentioning that the volume fraction of the α phase is higher after furnace cooling than after water quenching. The average volume fraction of the α phase after furnace cooling and after water quenching was found to be 78.71% and 69.42%, respectively.

#### 3.1.3. Microstructure of Supertransus Heat Treatments

**Figure 22:**
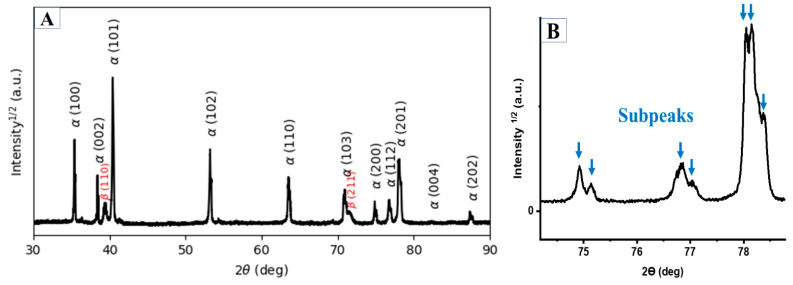
(**A**) The XRD pattern of the HT1020FC sample indexing α and β phases. (**B**) Enlargements of the (200), (112), and (201) peaks from 74° to 80° diffraction angles, indicating the formation of subpeaks.

**Figure 23:**
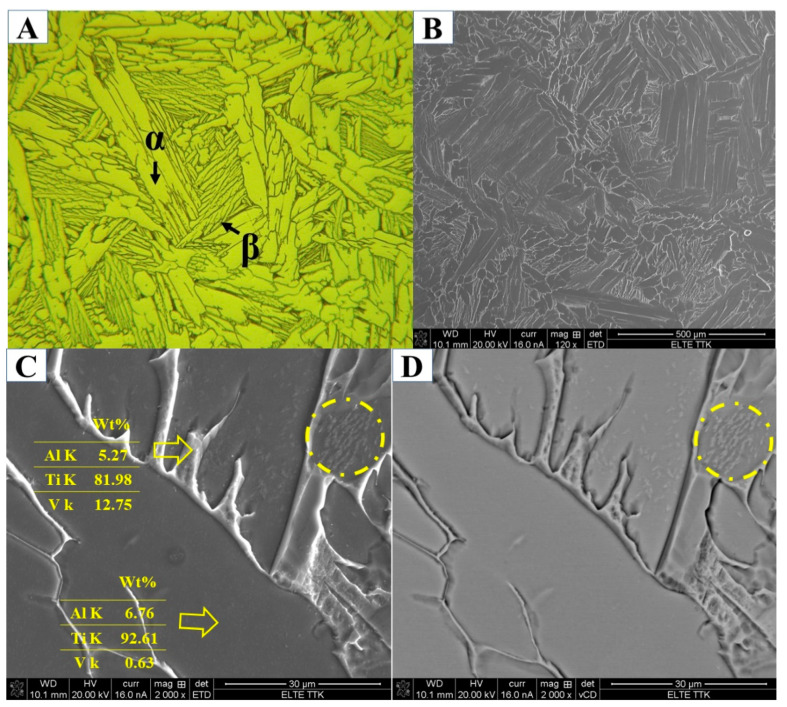
(**A**–**D**) SEM and optical micrographs showing microstructure of the HT1020FC sample at various magnifications.

Figure 22A presents the XRD patterns of the HT1020FC specimen, in which reflections related to α and β phases are identified. It is interesting to note that some reflections, such as (200), (112), and (201), consist of a series of subpeaks, as shown in Figure 22B. According to the literature, these findings demonstrate that α phases are precipitated at different temperatures with the same a and different c lattice parameters [31]. At the same time, the chemical composition and morphology of the α phases precipitated at different temperatures are also different. Figure 23 shows the optical and SEM images of Ti64 microstructure after HT1020FC heat treatment, revealing a lamellar microstructure of α and β phases. Figure 23C shows the chemical composition of these phases. As can be seen, the β phase is rich in V (12.75 wt.%) compared to the α phase (0.63 wt.%). The mean amount of the β phase after HT1020FC is about 9.14 ± 1 wt.%. Peak temperatures above β_tr_ occur during this heat treatment, which convert the α′ microstructure to 100% β. Owing to the slow cooling rate, β is subsequently converted to the dual-phase microstructure of α and β. It is noteworthy that some grains of the α phase exhibit particles of the β phase, as highlighted by a yellow circle in Figure 23C,D. The presence of β particles was detected in the heat treatment of as-manufactured Ti64 at 1150 °C for 2 h, followed by air cooling [38]. The hardness values of the HT 1020 FC averaged at 342 HV, which is lower than the hardness of the as-manufactured specimen. This is due to the decomposition of α′ martensite as well as the grain coarsening of the α phase during the heat treatments.

**Figure 24:**
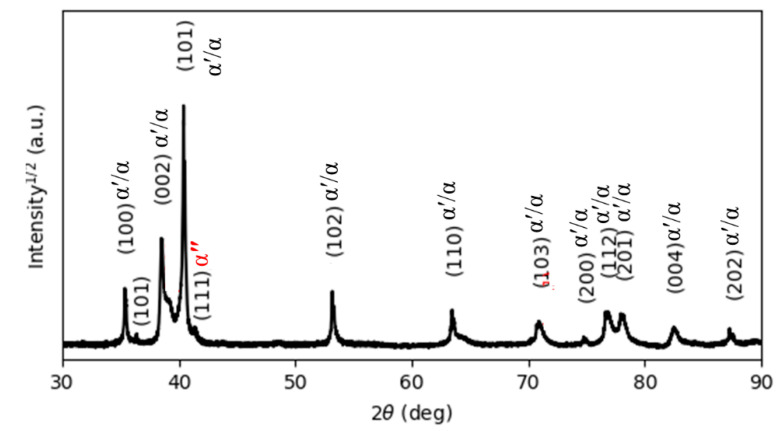
The XRD pattern of the HT1020WC sample indexing α′, α, and α″ phases.

**Figure 25:**
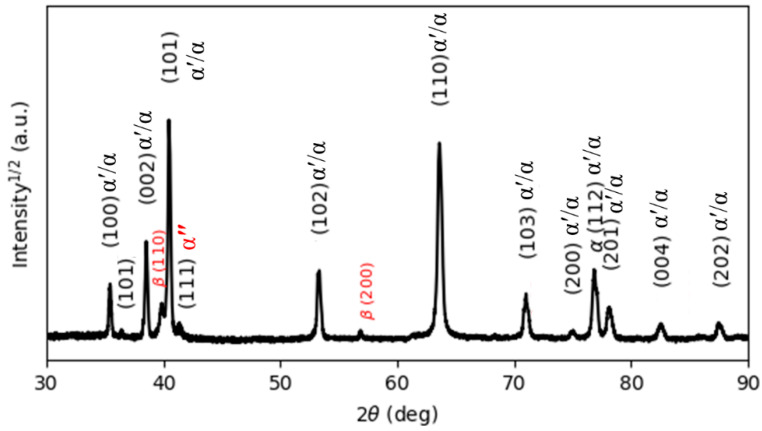
The XRD pattern of the HT1020WC + AG sample indexing α′, α, β, and α″ phases.

**Figure 26:**
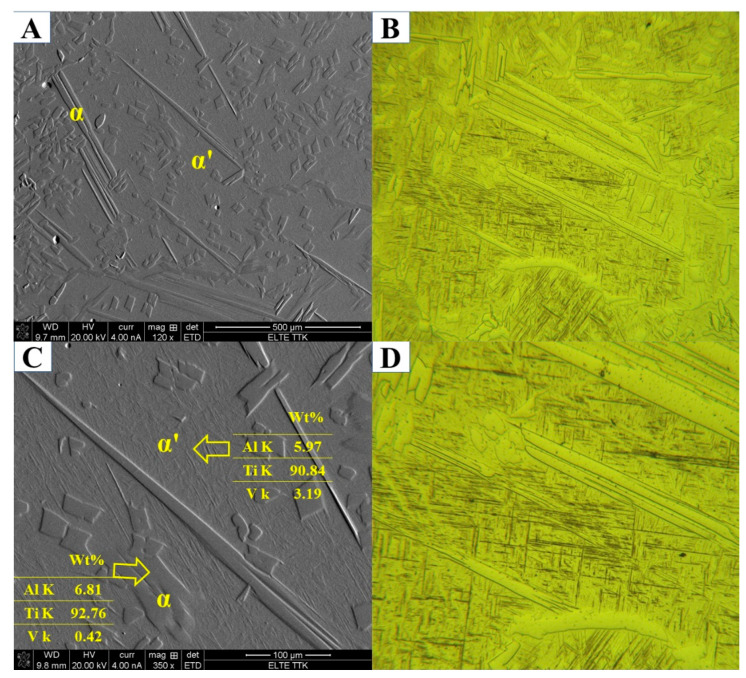
(**A**–**D**) SEM and optical micrographs showing microstructure of the HT1020WC sample at various magnifications.

The microstructure analysis of HT1020WC specimens after XRD measurements (Figure 24) showed the presence of α′, α, and α″ phases. No evidence of the β phase was found. In contrast, α′, α, β, and α″ phases were revealed in the HT1020WC + AG specimen (Figure 25). Moreover, the intensity of the (110) peak is significantly higher in the HT1020WC + A specimen than in HT1020WC. Both α and α′ phases exhibit a hexagonal structure. Therefore, it is difficult to distinguish between α and α′ phases by XRD. One of the most important metallurgical differences between α and α′ phases is the content of the V element. The content of V in the α′ phase is higher than that in the α phase, which is due to rapid cooling that prevents the diffusion of vanadium. Figure 26 shows the SEM and optical micrographs of the HT1020WC specimen at different magnifications. As can be seen, the EDS analysis (Figure 26C) reveals that the α′ phase is rich in V (3.19 wt.%) compared to the α phase (0.42 wt.%). The formation of α and α′ phases instead of α′ martensite is surprising. According to previous studies [39], the β phase formed when heated above the β_tr_ temperature is converted to a complete α′ martensitic phase when quenched with water. However, the reason is probably that the β_tr_ of the powder can be higher than 1020 °C. The higher hardness of HT1020WC (~379 HV) compared to the as-manufactured specimen (~377 HV) is due to the formation of the α′ martensite plus α phase.

**Figure 27:**
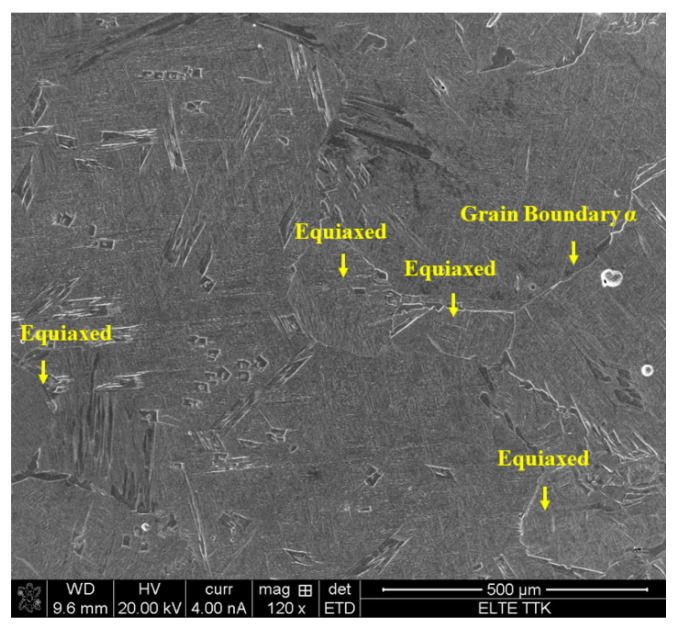
SEM micrograph showing semi-equiaxed β grain morphology of the HT1020WC sample.

In contrast to the HT1020FC process, where the formation of α + β lamellae is the main metallurgical feature (Figure 23), in the HT1020WC process, the prior columnar β structure of the as-manufactured Ti64 is replaced by semi-equiaxed β grains with a diameter of about 170 µm with longer elongated α grains and α′, as shown in Figure 27. The β phase undergoes more complicated microstructural transformations during solidification after HT1020WC, such as the formation of the α′ phase by the displacing transformation via a shear mechanism and the formation of the semi-equiaxed β grain morphology via a nucleation process.

**Figure 28:**
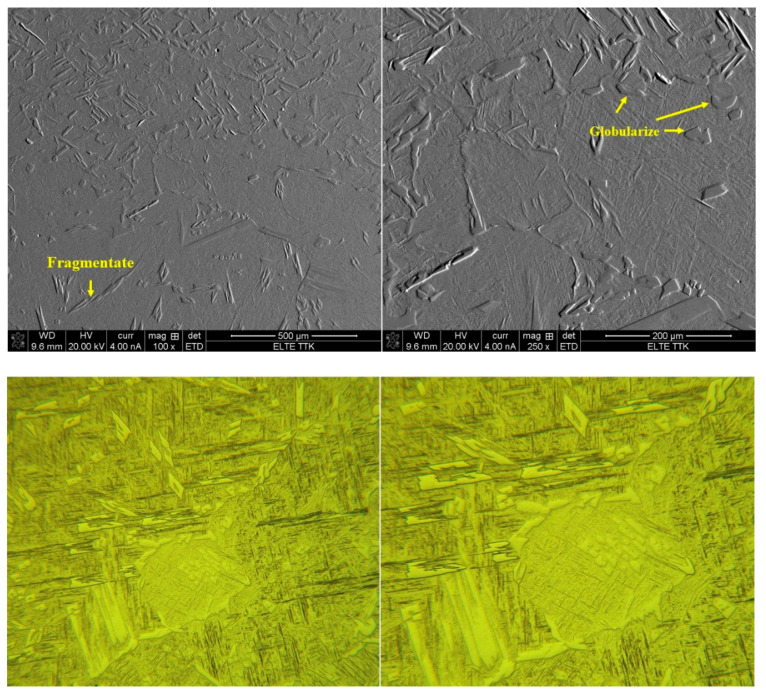
SEM and optical micrographs showing microstructure of the HT1020WC + AG sample at various magnifications.

**Figure 29:**
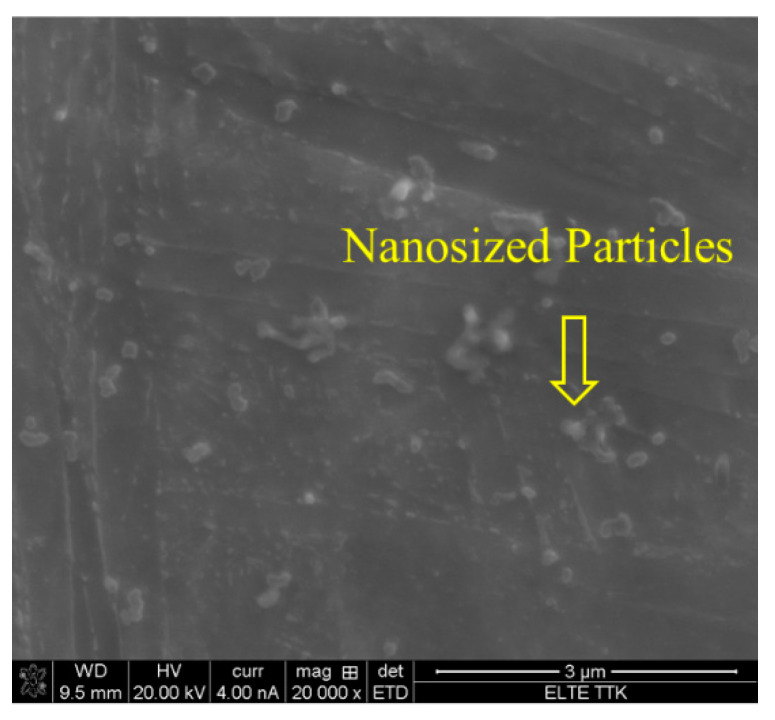
SEM micrographs at higher magnifications showing microstructure of the HT1020WC + AG sample and indicating the formation of β nanosized particles dispersed on the α phase.

The effect of aging (550 °C/3 h/FC) on the microstructure of HT1020WC specimens is summarized in Figure 28 at various magnifications. It can be seen that the longer elongated α grains have started to fragment and globularize, as indicated by the arrows in Figure 28. This result leads us to conclude that aging is the key factor in determining the final dimensions and the morphology of the α phase. The formation of the β phase after HT1020 WC + AG was not revealed by optical and SEM results. In contrast, the XRD result after HT1020 WC + AG indicates the formation of a small amount of β phase (Figure 25). The microhardness results confirm the SEM result of HT1020 WC +AG, as there is a slight increase after aging (384 HV instead of 379 HV for the HT1020WC specimen). Figure 29 shows the microstructure of the HT1020 WC + AG specimen at a higher magnification. It can be seen that some nanoscale particles are dispersed on the α phase.

### 3.2. Tensile Properties

The results of the mechanical properties obtained from the tensile test of as-manufactured and heat-treated specimens are summarized and compared in Table 3. As expected, the as-manufactured specimen has a high yield (1060 MPa) and a high ultimate tensile strength (1180 MPa) but a low ductility (8%) of less than 10%. This is due to the formation of a fine α′ martensitic microstructure. Interestingly, the ductility of all heat-treated SLM specimens is higher than that of the as-manufactured specimen, except for specimen (HT1020WC + AG). On the other hand, the strength of all heat-treated SLM specimens is lower than that of the as-manufactured specimen. This might be explained by the complete decomposition of α′ and the coarsening of the microstructure of heat-treated specimens compared to the original fine α′ martensite.

The evolution of mechanical properties during heat treatment of SLM parts is controlled by the decomposition of the α′ martensitic microstructure. It is important to note that HT850FC produces the best possible combination of ductility (13%) and strength properties (σ_y_ = 932, σ_u_ = 986 MPa) among all treatments, and the microstructure comprises β and α phases. This is due to the formation of the β phase in Ti64 alloy, resulting in a decrease in tensile strength and an increase in ductility. From the lower strength of HT850WC (σ_y_ = 870, σ_u_ = 930 MPa) and HT850WC + AG (σ_y_ = 892, σ_u_ = 970 MPa) compared to HT850FC (σ_y_ = 932, σ_u_ = 986 MPa), it could be interpreted that the soft martensitic α″ microstructure is formed in prior β grains during rapid solidification of the β phase upon water quenching.

It has been found that the mechanical properties of Ti alloys are a function of the thickness of α lath and α colony as well as grain boundary α [40]. A larger α lath and the complete degradation of α′ martensite may explain the lower strength of treatment at HT1020FC (σ_y_ = 748, σ_u_ = 833 MPa) in comparison with HT850FC. The average lath thickness of the α phase was 10.63 µm after HT1020FC compared to 1.403 µm after HT850FC and 0.420 µm after printing.

HT1020WC exhibits high strength (σ_y_ = 878, σ_u_ = 990 MPa) and low ductility (8.6) compared to all heat treatment processes. At the same time, the tensile strength of the HT1020WC specimens decreases compared to the as-manufactured specimens, but the ductility does not increase. This is due to the presence of 27.33 vol.% of lamellar α and 72.67 of α′ in the microstructure instead of complete α′, which leads to a decrease in strength. Another possible reason is that the lath thickness of α (13.6 µm) in HT1020WC specimens is higher than that in the as-manufactured and other heat treatments specimens, which can reduce the mechanical properties.

Interestingly, the strength of HT850 WC + AG (σ_y_ = 944, σ_u_ = 1035 MPa) is slightly higher than that of HT1020WC, but its ductility is lower (7.2%). The reason is probably the presence of some nanoscale β particles distributed on α′, which, in turn, increases the strength and decreases the ductility.

## 4. Conclusions

1. In the microstructure of Ti64, a very fine acicular martensite α′ with a small amount of β and α″ structure developed due to the extremely high cooling rate associated with the SLM. Microstructural observations confirmed the complete decomposition of the fine acicular martensite α′ during the post-heat treatment cycle, the transformation of α′ to α, β, and α″ phases, and the formation of some nanoscale β particles during the cooling stage, confirming the need for post-treatments after SLM of Ti64.
2. The best mechanical properties were obtained by heat treatment at 850 °C followed by cooling in the furnace. This heat treatment enhanced the ductility to 13%, compared to 8% for as-manufactured specimens. The improved ductility of HT850FC can be attributed to the complete decomposition of α′ into mainly α plus β and a small amount of α″ phases as well as the coarsening of the microstructure of HT850FC compared to the original fine α′ martensite.
3. No improvement in mechanical properties was observed for HT850WC due to the formation of soft orthorhombic α″, α, and β. The presence of α″ is responsible for a significant decrease in hardness.
4. The microstructure of HT1020FC is characterized by the formation of an α + β lamellar structure. In contrast, the microstructure of HT1020WC is characterized by the formation of semi-equiaxial β grains with a diameter of about 170 µm with longer elongated α grains and basket-weave α′. Moreover, XRD analysis confirmed the presence of α″.

## Figures and Tables

**Table 1:** Chemical analysis of Ti64 powder and ASTM specification.

(Mass%)		Al	V	Fe	O	N	C	H	Ti
Ti6Al4V powder		6.11	4.02	0.17	0.090	0.023	0.01	0.003	Bal
ASTM B348 Gr.23	Max	6.50	4.50	0.25	0.13	0.03	0.08	0.0125	Bal
	Min	5.50	3.50	-	-	-	-	-	-

**Table 2:** Lattice parameters and FWHM of the main α/α′ and β peaks at 2θ = 40.56° and 39.80°, respectively, of as-printed sample and samples subjected to different heat treatments.

Process	α/α′ Phase					β Phase		
	Lattice Parameters (Å)			(101)		Lattice Parameters (Å)	(110)	
Sample Name	a	c	c/a	2θ°	FWHM	a	2θ°	FWHM
As-printed	2.9324	4.6716	1.5930	40.56	0.1466	-	-	-
HT850FC	2.9218	4.6667	1.5972	40.54	0.0876	3.1989	39.80	0.253
HT850WC	2.9236	4.6701	1.5973	40.51	0.094	3.2419	39.25	0.27
HT850WC + AG	2.9228	4.6692	1.5975	40.52	0.0792	3.2001	39.80	0.211
HT1020FC	2.9264	4.6819	1.5956	40.46	0.1008	3.2316	39.41	0.366
HT1020WC	2.9295	4.6699	1.5968	40.43	0.114	-	-	-
HT1020WC + AG	2.9238	4.6714	1.5977	40.47	0.1373	3.1973	39.80	0.243

**Table 3:** Mechanical properties of the as-printed sample and samples subjected to different heat treatments.

No.	T/°C	t/h	Cooling Rate	Aging			YS/MPa	UTS/MPa	T.E%
				T/°C	t/h	Cooling			
1							1060	1180	8
2	850	2	FC	-	-	-	932	986	13
3	850	2	WC	-	-	-	870	930	10.4
4	850	2	WC	550	3	FC	892	970	9.3
5	1020	1	FC	-	-	-	748	833	14.5
6	1020	1	WC	-	-	-	878	990	8.6
7	1020	1	WC	550	3	FC	944	1035	7.2

T.E: total elongation.

## Data Availability

The data will be made available upon request.
